# Development and Validation of a Stability-Indicating RP-HPLC Method for the Estimation of Drotaverine Impurities in API and Pharmaceutical Formulation

**DOI:** 10.3797/scipharm.1309-06

**Published:** 2013-10-21

**Authors:** Veera Raghava Raju Thummala, Satya Sankarsana Jagan Mohan Tharlapu, Vijay Kumar Rekulapalli, Mrutyunjaya Rao Ivaturi, Someswara Rao Nittala

**Affiliations:** 1 Analytical Research and Development, Integrated Product Development, Dr. Reddy’s Laboratories Ltd., Bachupally, Hyderabad-500 072, India.; 2 School of Chemistry, Andhra University, Visakhapatnam-530003, A.P., India.

**Keywords:** Drotaverine, RP-HPLC, Stability-indicating, Impurities, ICH guidelines

## Abstract

A sensitive, stability-indicating gradient RP-HPLC method with PDA detection has been developed for the simultaneous analysis of drotaverine impurities in active pharmaceutical ingredient (API) and pharmaceutical formulations. Efficient chromatographic separation was achieved on an XTerra RP18, 150 × 4.6 mm, 5 μm column using gradient elution at 230 nm detection wavelength. The optimized mobile phase consisted of a 0.02 M potassium dihydrogen orthophosphate buffer of pH 3.0 as solvent A and acetonitrile as solvent B. The flow rate of the mobile phase was 1.0 mL min^−1^ with a column temperature of 25°C. The method showed linearity over the range of 0.251–10.033 μg/mL, 0.231–9.995 μg/mL, 0.230–10.089 μg/mL, 0.334–10.011 μg/mL, and 0.324–10.050 μg/mL for impurities 1, 2, 3, 4, and drotaverine, respectively, with a correlation coefficient greater than 0.999. The relative retention times and relative response factors of impurities 1, 2, 3, 4 were 0.36, 0.90, 1.42, 1.55 and 1.04, 0.84, 1.10, 1.30, respectively. The drotaverine formulation sample was subjected to the stress conditions of acid, base, oxidative, thermal, humidity, and photolytic degradation. Drotaverine was found to degrade significantly in peroxide, base, and heat stress conditions. The degradation products were well-resolved from drotaverine and its impurities. The peak purity test results confirmed that the drotaverine peak was homogenous and pure in all stress samples and the mass balance was found to be more than 98%, thus proving the stability-indicating power of the method. The developed method was validated according to ICH guidelines with respect to specificity, linearity, limit of detection and quantification, accuracy, precision, and robustness.

## Introduction

**Fig. 1:**
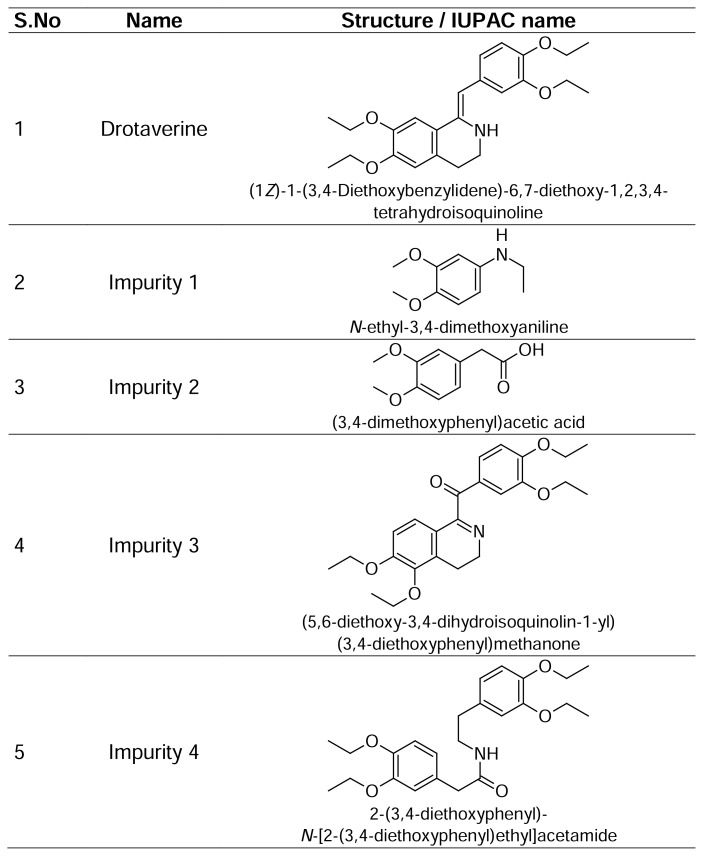
Chemical structures of drotaverine and its impurities

Drotaverine (Fig. 1) is chemically known as 1-(3,4-diethoxybenzylidene)-6,7-diethoxy-1,2,3,4-tetrahydroisoquinoline [1]. Drotaverine hydrochloride is a highly potent spasmolytic agent [2]. It acts as an antispasmodic agent by inhibiting the phosphodiesterase IV enzyme, specific for smooth muscle spasms and pain, and used to reduce excessive labor pains [3].

The impurity profile of API and pharmaceutical formulations is one of the most challenging tasks of pharmaceutical analytical chemists under industrial environmental conditions [4]. The presence of unwanted or in certain cases unknown chemicals, even in small amounts, may influence not only the therapeutic efficacy, but also the safety of the pharmaceutical products [5]. For these reasons, all major international pharmacopoeia have established maximum allowed limits for related compounds for both bulk and formulated APIs. As per the requirements of various regulatory authorities, the impurity profile study of drug substances and drug products has to be carried out by using a suitable analytical method in the final product [6, 7].

A detailed literature survey revealed that there are some analytical methods reported for the estimation of drotaverine either individually or in combination with other drugs like HPTLC [8], spectrophotometric [9–14], by derivative spectrophotometry [15], and by HPLC [16–32]. The route of synthesis of drotaverine and possible degradants resulted in four known impurities, Impurity 1, Impurity 2, Impurity 3, and Impurity 4, which are not reported in any of the pharmacopeia.

To date, there is not a single method that has been reported for the determination of the impurities either in bulk drugs or in pharmaceutical formulations of drotaverine. It is necessary to develop a stability-indicating method for drotaverine-related impurities in API and tablet dosage formulation.

Hence, an attempt has been made to develop an accurate, rapid, specific, and reproducible method for the determination of drotaverine impurities (Fig. 1) in API and in pharmaceutical dosage forms along with method validation as per ICH guidelines [33, 34]. The stability tests were also performed on the drug substance and drug product as per ICH guidelines [35, 36].

## Experimental

### Chemicals, Reagents, and Samples

Drotaverine tablets were received from formulation research and the development laboratory of Dr. Reddy’s Laboratories Ltd., IPDO, Hyderabad, India. Drotaverine API and impurities were procured from RA Chem Ltd., India. Potassium dihydrogen orthophosphate was procured from Merck, Germany. HPLC grade acetonitrile and orthophosphoric acid were purchased from Merck, Germany, and high purity water was prepared by using the Millipore Milli-Q Plus purification system.

### Equipment

A Waters HPLC system with a DAD (2996 detector and 2695 separation module with quaternary gradient) was used for method development and method validation. The output signal was monitored and processed using Waters Empower software. Weighing was performed with a Mettler XS 205 dual range (Mettler-Toledo GmbH, Greifensee, Switzerland). Photostability studies were carried out in a photostability chamber (SUN TEST XLS+, Atlas, USA). Thermal stability studies were performed in a dry air oven (Merck Pharmatech, Hyderabad, India).

### Chromatographic System

HPLC measurements were carried out using a reversed-phase XTerra RP 18, 150 × 4.6 mm, 5 μ particle size column (Waters) operated at 25°C with gradient elution at 1.0 mL min^−1^; UV absorbance at 230 nm; injection volume 20 μL. Mobile phase A consisted of a 3.0 pH 0.02 M potassium dihydrogen orthophosphate buffer and acetonitrile (90:10 v/v); mobile phase B consisted of a pH 3.0 pH 0.02 M potassium dihydrogen orthophosphate buffer and acetonitrile (40:60 v/v). The LC gradient program was set as: time (min)/% mobile phase B: 0.01/30, 7.5/80, 15/90, 17/30, and 20/30. Water and acetonitrile (70:30 v/v) were used as diluent for the sample preparation.

### Preparation of Standard Solution and System Suitability Solution

A stock solution of drotaverine (1000 μg mL^−1^) was prepared by dissolving an appropriate amount in diluent. Working solution was prepared from the above stock solution for related substances’ determination (2 μg mL^−1^ of drotaverine) in diluent. A mixture of all impurities (2.0 μg mL^−1^) along with drotaverine (1000 μg mL^−1^) was prepared in diluent. Also impurity stock solutions were prepared in diluent.

### Preparation of Test Solution

Twenty tablets’ (drotaverine label claim: 80 mg per tablet) content was weighed and the average weight of each tablet was calculated. Tablet powder equivalent to 100 mg of the active pharmaceutical ingredient (drotaverine) was transferred into a 100 mL volumetric flask. To this, 70 mL of diluent was added and sonicated for 30 minutes with intermediate shaking. The solution was then diluted to 100 mL with diluent and centrifuged at 3000 rpm for 10 min. The supernatant (1000 μg mL^−1^ of drotaverine) was collected and used as sample solution.

## Method Validation

The proposed method was validated as per ICH guidelines [34].

### System Suitability

System suitability parameters were performed to verify the system performance. The system precision was determined on six replicate injections of the standard preparation. All of the important characteristics, including the relative standard deviation (RSD), peak tailing, and theoretical plate number, were measured. The resolutions between the impurities were measured by injecting the system suitability solution. All of these system suitability parameters covered the system, method, and column performance.

### Specificity

Stress studies were performed at an initial concentration of 1000 μg mL^−1^ of drotaverine in API and a formulated sample to provide the stability-indicating property and specificity of the method. Intentional degradation was attempted by the stress conditions when exposed to acid (0.1 N HCl for 20 min at 60°C), base (0.1 N NaOH for 20 min at 60°C), oxidation (3% hydrogen peroxide for 20 min at 60ºC), heat (exposed at 105°C for 3 h), humidity (exposed to 90% RH for 7 days), and photolytic stress (1.2 million lux hours followed by 200 watt hours m^−2^).

### Precision

The precision for the determination of the impurities was checked by injecting six individual preparations of drotaverine (1000 μg mL^−1^) test preparations spiked with 2.0 μg mL^−1^ of Impurity 1, Impurity 2, Impurity 3, and Impurity 4, and calculated the RSD of each impurity content and retention time. The intermediate precision of the method was also evaluated using different analysts and a different instrument in the same laboratory on a different day. Also, the precision and intermediate precision study was performed by spiking 2.0 μg mL^−1^ of drotaverine on the placebo as per test preparation.

### Accuracy

The accuracy of the method was demonstrated at five different concentration levels in triplicate. The analysis was carried out by spiking all of the impurities on the formulation sample at 0.1, 0.2, 0.5, 0.75, and 1.0% of the drotaverine concentration (1000 μg mL^−1^). Also, the accuracy study was performed by spiking drotaverine on the placebo at the above-mentioned levels. The percentage mean recoveries at each level for all of the impurities and drotaverine were calculated.

### Limit of Detection (LOD) and Limit of Quantification (LOQ)

The LOD and LOQ for Impurity 1, Impurity 2, Impurity 3, Impurity 4, and drotaverine were estimated at a S/N of 3:1 and 10:1, respectively, by injecting a series of dilute solutions with known concentrations. The precision and accuracy studies were also carried out at the LOQ level.

### Linearity

The linearity solutions were prepared from stock solutions at six concentration levels from the LOQ to 1.0% of the analyte concentration. The peak area versus concentration data were subjected to least-squares linear regression analysis. The calibration curve was drawn by plotting impurity areas against the concentration expressed in μg mL^−1^.

### Robustness

To determine the robustness of the developed method, experimental conditions were deliberately changed and the resolution between drotaverine & its impurities, tailing factor, and theoretical plates of the drotaverine peak were evaluated.

To study the effect of the flow rate on the developed method, it was changed from 1.0 mL min^−1^ to 0.8 and 1.2 mL min^−1^. The effect of column temperature on the developed method was studied at 20 and 30°C (instead of 25°C). The effect of pH was studied by varying ± 0.2 pH units (i.e. 2.8 and 3.2) and the mobile phase composition was changed ±10% from the initial composition. In all of the above-varied conditions, the component of the mobile phase was held constant.

### Stability in Solution and in the Mobile Phase

Drotaverine-spiked samples (impurities spiked at 0.2% of the analyte concentration) were prepared in the diluent, leaving the test solutions at room temperature. The spiked samples were injected at 0, 24, 48 hrs time intervals. The impurity content was calculated, and the consistency in the content of each impurity at each interval was checked. The prepared mobile phase was kept constant during the study period. The mobile phase study was demonstrated by injecting the freshly prepared sample solution at different time intervals (0–2 days).

## Results and Discussion

### Optimization of Chromatographic Conditions

The main criterion for developing an RP-HPLC method for the determination of impurities in drotaverine pharmaceutical dosage form was to be in a single run, with emphasis on the method being accurate, reproducible, robust, stability-indicating, linear, free of interference from other formulation excipients, and convenient enough for routine use in quality control laboratories.

**Fig. 2:**
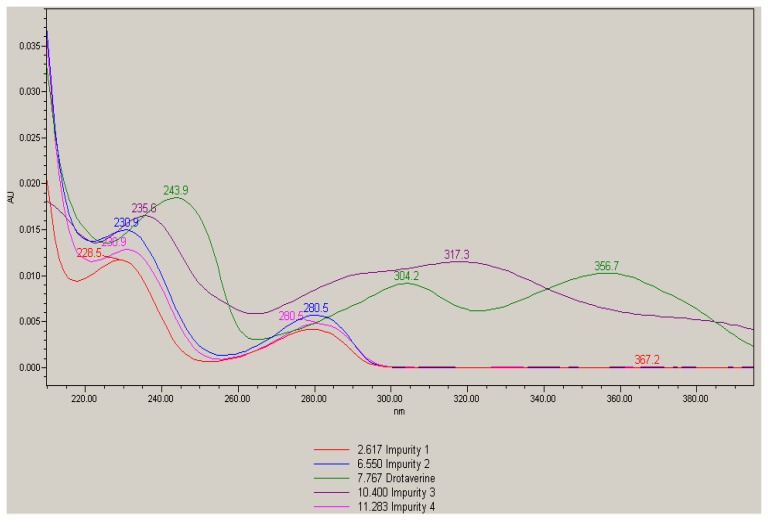
Overlay spectras of drotaverine and its impurities

Individual stock solutions of drotaverine and its impurities were scanned in a photodiode array detector in the range of 200 to 400 nm and checked the spectra of each component. From the spectra (Fig. 2), all the impurities had an absorbance maximum at about 230 nm. Hence 230 nm was selected for the estimation of drotaverine impurities.

The drotaverine sample preparation (1000 μg mL^−1^) spiked with all the impurities (2.0 μg mL^−1^) and placebo preparation was subjected to separation by RP-HPLC. Initially, the separation of all the peaks was studied by using a reversed-phase XTerra RP18, 150 × 4.6 mm, 5 μ particle size column with isocratic elution. The mobile phase consisted of a 0.02 M potassium dihydrogen orthophosphate buffer of pH 3.0 (pH-adjusted with dilute orthophosphoric acid solution) and acetonitrile in the ratio of 50:50 (v/v). A 1.0 mL/min flow rate was selected to achieve the separation of all the peaks and the column oven temperature was maintained at 25°C. It was observed that impurities 1, 2, and a few unknown peaks eluted close to the void volume with improper separation. It was also observed that one unknown impurity peak was eluted at the retention time of 25 minutes. To achieve separation between the early eluting peaks, the buffer concentration was increased to 70% instead of 50%, but the separation was not up to the mark and one unknown impurity peak eluted at 50 minutes. Separation was tried further by decreasing the flow rate, which resulted in a longer run time.

Based on above experiments, the isocratic program was replaced with the gradient program in an effort to achieve high resolution between the known impurities and all degradant peaks within a shorter run time. With the same column, different combinations of mobile phase A and B were studied with different gradient programmes to optimize the method. Finally, the chromatographic separation was achieved and the finalized conditions were mentioned in the chromatographic system under the “Experimental” section. All the impurities were well-separated with a resolution greater than 2, typical retention times of drotaverine, Impurity 1, Impurity 2, Impurity 3, and Impurity 4 were 7.360 min, 2.664 min, 6.617 min, 10.447 min, and 11.382 min, respectively. No chromatographic interference due to the blank (diluent) and other excipients (placebo) at the retention time of drotaverine and all impurities were observed. The typical overlay chromatogram of the blank and system suitability solution, placebo, and spiked sample is shown in Fig. 3a & 3b.

### Response Factor

The measurement of the response factor for each impurity determination is important when the calculations are being made on a relative percent basis. Relative response factor was calculated from the ratio of the slope of each impurity against the slope of the drotaverine standard. Hence an authentic sample of drotaverine and its impurities were dissolved in the diluent and prepared series of solutions. The details of solution preparations were mentioned in the “Linearity” section under “Experimental.” The slope and response factor values are mentioned in Table 6.

## Method Validation

After the development of the method, it was subject to method validation as per ICH guidelines [34]. The method was validated to demonstrate that it is suitable for its intended purpose by the standard procedure to evaluate the adequate validation characteristics (system suitability, specificity, accuracy, precision, linearity, robustness, ruggedness, solution stability, LOD, LOQ, and stability-indicating capability).

### System suitability

The percentage relative standard deviation (RSD) of the area from six replicate injections was below 5.0% (diluted standard solution, 2.0 μg mL^−1^ of drotaverine). Low values of the RSD for replicate injections indicate that the system is precise. The results of other system suitability parameters such as resolution, peak tailing, and theoretical plates are presented in Table 1. As seen from this data, the acceptable system suitability parameters would be as follows: the relative standard deviation of replicate injections is not more than 5.0%, resolution between the impurities is 2.5, the tailing factor for drotaverine is not more than 1.5, and the theoretical plates are not less than 10000.

### Specificity

All forced degradation samples were analyzed with the aforementioned HPLC conditions using a PDA detector to monitor the homogeneity and purity of the drotaverine peak and its related impurities. Individual impurities, the placebo, and drotaverine were verified and proved to be non-interfering with each other, thus proving the specificity of the method.

Figure 3b shows that there is no interference at the RT (retention time) of drotaverine and all known impurities from the other excipients. Degradation was not observed in the acid stress, humidity stress, and photo stress studies. Significant degradation was observed in the base stress, peroxide stress, and heat stress studies. It is interesting to note that all the peaks were well-resolved due to degradation from the peaks of drotaverine and its impurities. Further, the peak purity of drotaverine and its impurities was found to be homogeneous based on the evaluation parameters such as purity angle and purity threshold using Waters Empower Networking Software. The verification of peak purity indicates that there is no interference from the degradants, facilitating error-free quantification of the drotaverine impurities. Also, the mass balance of the stressed samples was found to be more than 98%. Thus, the method is considered to be “stability-indicating”. The specificity results are shown Table 2 and base, peroxide, and heat stress chromatograms are shown in Figs. 4a, 4b & 4c.

### Precision

In the method precision study, the RSD was within 0.5 for Impurity 1, 1.0 for Impurity 2, 1.6 for Impurity 3, 0.7 for Impurity 4, and 0.5 for drotaverine, respectively. In the intermediate precision study, the RSD was within 2.4 for Impurity 1, 2.6 for Impurity 2, 2.5 for Impurity 3, 1.3 for Impurity 4, and 2.4 for drotaverine, respectively. Also, the RSD for the retention times for each component from both the precision and intermediate precision study were within 0.3. The results are shown in Tables 3a & 3b.

### Accuracy

The recovery of all of the four impurities and drotaverine from the finished pharmaceutical dosage form ranged from 85.0% to 115.0%. The summary of % recovery for each individual impurity is mentioned in Table 4.

### Limit of Detection (LOD) and Limit of Quantification (LOQ)

LOD values were achieved at 0.076, 0.083, 0.110, 0.076, and 0.107 μg mL^−1^ for Impurity 1, Impurity 2, Impurity 3, Impurity 4, and drotaverine, respectively. LOQ values were achieved at 0.231, 0.251, 0.334, 0.230, and 0.324 μg mL^−1^ for Impurity 1, Impurity 2, Impurity 3, Impurity 4, and drotaverine, respectively. The % RSD of precision at the LOQ concentration for Impurity 1, Impurity 2, Impurity 3, Impurity 4, and drotaverine was found to be below 5.0. The results of precision at the LOQ level is shown in Table 5.

### Linearity

Linearity regression analysis demonstrated the acceptability of the method for the quantitative determination range of the LOQ (0.231, 0.251, 0.334, 0.230, and 0.324 μg mL^−1^ for Impurity 1, Impurity 2, Impurity 3, Impurity 4, and drotaverine, respectively) to 10 μg mL^−1^. The correlation coefficient was found to be more than 0.997. The regression statistics are shown in Table 6.

### Robustness

No significant effect was observed on the system suitability parameters such as resolution, RSD, tailing factor, or the theoretical plates of drotaverine when small, but deliberate changes were made to the chromatographic conditions. The results are presented in Table 1, along with the system suitability parameters of normal conditions. Thus, the method was found to be robust with respect to variability in applied conditions.

### Stability in Solution and in the Mobile Phase

No significant changes were observed in the content of impurities, namely Impurity 1, Impurity 2, Impurity 3, and Impurity 4 during the solution stability and mobile phase stability experiments when performed using the impurities method. The solution stability and mobile phase stability experiment data confirm that the sample solution and mobile phases used during the impurity determination were stable for at least 48 h.

## Conclusion

The gradient HPLC method developed for the determination of drotaverine impurities in both bulk drug and pharmaceutical dosage form was precise, accurate, and specific. The method is validated as per ICH guidelines and found to be specific, precise, linear, accurate, rugged, and robust. The developed method can be used for the stability analysis of both drotaverine API and formulated samples.

## Figures and Tables

**Fig. 3a:**
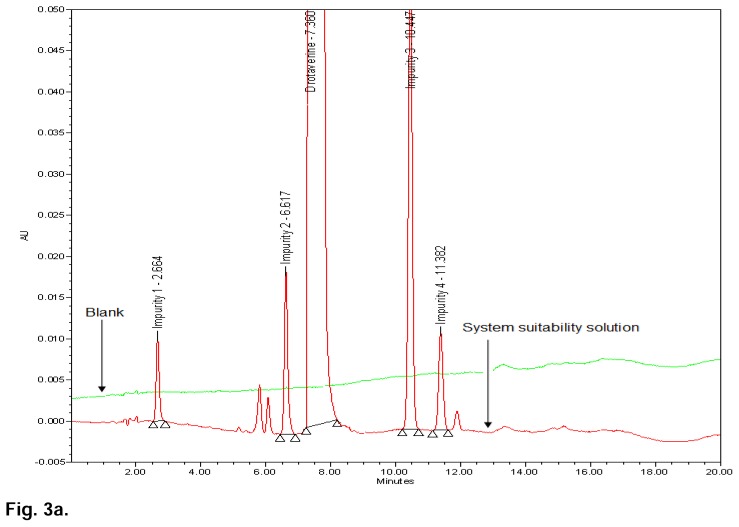
HPLC overlay chromatograms of blank and system suitability solution

**Fig. 3b:**
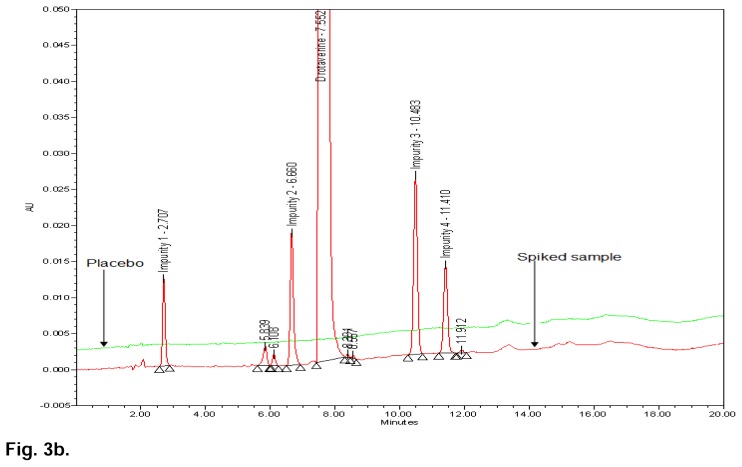
HPLC overlay chromatograms of placebo and spiked sample

**Fig. 4a:**
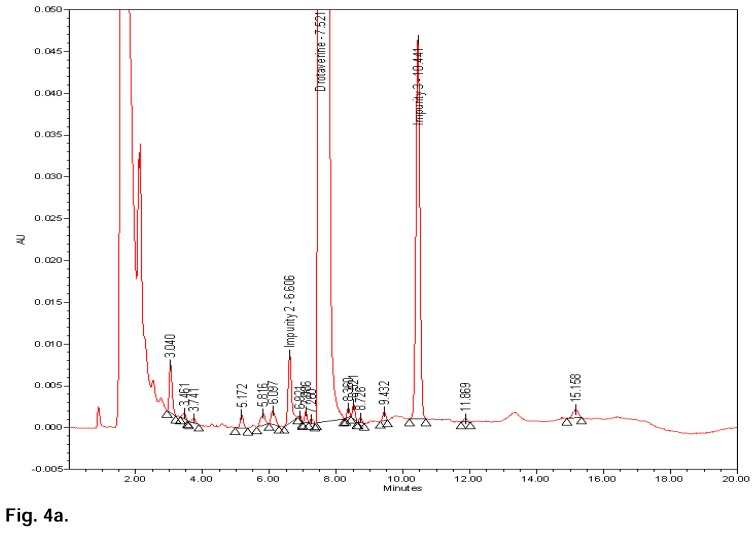
Typical chromatogram and purity plot of base-stressed sample

**Fig. 4b:**
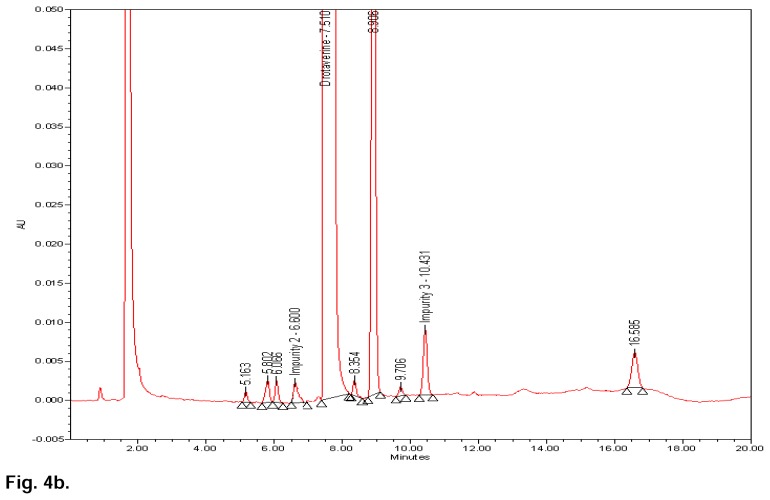
Typical chromatogram of peroxide-stressed sample

**Fig. 4c:**
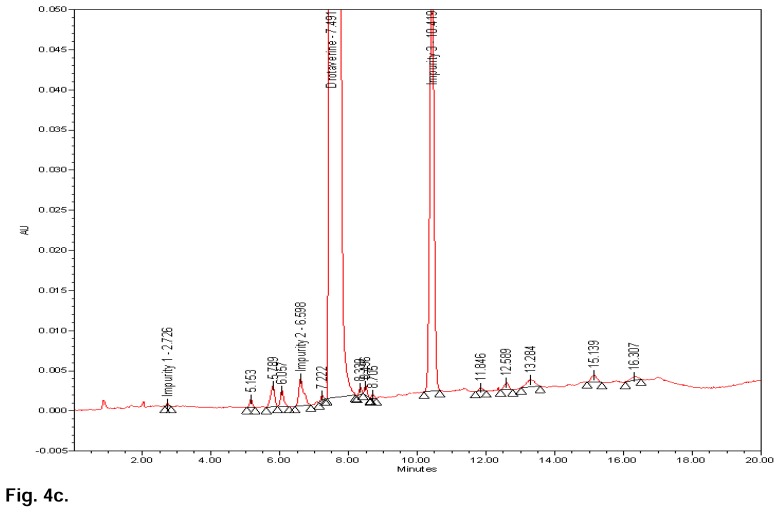
Typical chromatogram of heat-stressed sample

**Tab. 1:** System suitability results

Parameter	Peak area (RSD %)*	Theoretical plates*	Tailing Factor*	Resolution	

				1	2
As such method	0.6	32729	1.1	3.5	4.2
Low flow rate (0.8 ml/min)	0.4	29947	1.0	2.9	4.0
High flow rate (1.2 ml/min)	0.8	29838	1.0	2.2	3.9
Low column temperature (20°C)	0.2	33092	1.1	2.8	3.8
High column temperature (30°C)	0.5	32779	1.1	3.4	4.1
Low pH (2.8)	0.3	29712	1.0	3.1	3.7
High pH (3.2)	0.7	29878	1.0	2.8	3.5
Low Organic phase composition in mobile phase B (−10%)	0.6	23831	1.0	3.3	4.2
High Organic phase composition in mobile phase B (+10%)	0.5	33297	1.1	3.0	3.7

* Determined on six values.

Resolution 1: Resolution between drotaverine and Impurity 2.

Resolution 2: Resolution between Impurity 3 and Impurity 4.

**Tab. 2:** Forced degradation data for drotaverine

Degradation conditions	Drotaverine			

	% degraded	Purity angle	Purity Threshold	Mass balance (%)
Acid Stress	0.9	0.267	1.957	99.8
Base Stress	3.4	0.335	1.789	99.1
Peroxide Stress	6.7	0.258	1.570	100.2
Heat Stress	3.5	0.424	1.794	98.9
Humidity Stress	0.4	0.425	2.241	99.3
Photo Stress	0.6	0.344	1.883	99.2

**Tab. 3a:** Results of method precision (analyst 1, instrument 1, column 1, and day 1)

Preparation	Impurity 1	Impurity 2	Impurity 3	Impurity 4	Drotaverine
Prep-1	0.202	0.194	0.197	0.205	0.196
Prep-2	0.200	0.197	0.199	0.205	0.195
Prep-3	0.200	0.194	0.202	0.206	0.198
Prep-4	0.202	0.197	0.202	0.205	0.197
Prep-5	0.202	0.192	0.206	0.208	0.197
Prep-6	0.202	0.196	0.204	0.204	0.196
Avg	0.202	0.195	0.202	0.206	0.197
%RSD	0.5	1.0	1.6	0.7	0.5
% RSD for retention time	0.3	0.2	0.2	0.3	0.1

**Tab. 3b:** Results of intermediate method precision (analyst 2, instrument 2, column 2, and day 2)

Preparation	Impurity 1	Impurity 2	Impurity 3	Impurity 4	Drotaverine
Prep-1	0.199	0.208	0.198	0.212	0.197
Prep-2	0.203	0.210	0.200	0.210	0.209
Prep-3	0.195	0.199	0.203	0.215	0.205
Prep-4	0.193	0.201	0.210	0.207	0.199
Prep-5	0.202	0.199	0.198	0.213	0.201
Prep-6	0.205	0.210	0.208	0.210	0.197
Avg	0.200	0.205	0.203	0.211	0.201
%RSD	2.4	2.6	2.5	1.3	2.4
% RSD for retention time	0.1	0.2	0.1	0.2	0.2

**Tab. 4:** Accuracy of the method

Spike Level	Recovery (%)^a^				

	Impurity 1	Impurity 2	Impurity 3	Impurity 4	Drotaverine
0.1%	100.6±0.6	103.4±0.8	97.8±2.1	99.9±1.2	97.5±0.4
0.2%	100.1±0.4	101.4±1.6	102.7±0.2	97.9±1.4	99.5±0.9
0.5%	100.6±1.1	105.5±0.7	101.1±0.4	95.2±0.8	100.2±0.5
0.75%	100.9±0.9	104.7±1.3	102.6±0.7	95.7±0.4	98.5±0.5
1.0%	100.4±0.3	103.2±1.9	103.0±0.5	97.9±0.9	99.2±0.8

a Mean ± standard deviation for three determinations

**Tab. 5:** Results of precision at limit of quantification

Preparation	% of each impurity				

	Impurity 1	Impurity 2	Impurity 3	Impurity 4	Drotaverine
Prep-1	0.025	0.023	0.023	0.033	0.032
Prep-2	0.026	0.022	0.025	0.034	0.030
Prep-3	0.025	0.025	0.024	0.032	0.029
Prep-4	0.024	0.024	0.025	0.033	0.032
Prep-5	0.027	0.023	0.024	0.031	0.033
Prep-6	0.023	0.022	0.022	0.032	0.031
Avg	0.025	0.023	0.024	0.033	0.031
%RSD	0.7	5.0	4.9	3.2	4.7

**Tab. 6:** Regression statistics and relative response factor results

Substance	Linearity range (μg mL^−1^)	Correlation coefficient (R^2^)	Y-intercept Bias in %	Slope	Relative response factor
Impurity 1	0.251–10.033	1.000	3.0	39419.7	1.04
Impurity 2	0.231–9.995	1.000	1.7	31872.2	0.84
Impurity 3	0.230–10.089	1.000	1.8	41923.2	1.10
Impurity 4	0.334–10.011	1.000	1.8	49327.6	1.30
Drotaverine	0.324–10.050	1.000	1.5	37977.5	–
